# Identification of PLAUR-related ceRNA and immune prognostic signature for kidney renal clear cell carcinoma

**DOI:** 10.3389/fonc.2022.834524

**Published:** 2022-08-16

**Authors:** Yu Wang, Zhuolun Sun, Shuo Lu, Xu Zhang, Chutian Xiao, Tengcheng Li, Jieying Wu

**Affiliations:** ^1^ Department of Urology, The Third Affiliated Hospital, Sun Yat-sen University, Guangzhou, China; ^2^ Department of Gynecology, The Third Affiliated Hospital, Sun Yat-sen University, Guangzhou, China; ^3^ Department of Urology, The Sixth Affiliated Hospital, Sun Yat-sen University, Guangzhou, China

**Keywords:** ceRNA, immunomodulators, kidney renal clear cell carcinoma, PLAUR, prognostic signature

## Abstract

Kidney renal clear cell carcinoma (KIRC) represents one of the most fatal cancers, usually showing malignant progression and a high tumor recurrence rate. The urokinase-type plasminogen activator receptor (PLAUR) plays a critical role in the initiation and progression of several cancers, including KIRC. However, the function and mechanism of PLAUR in patients with KIRC are still unclear and require further investigation. In the present study, we first explored the expression profile and prognostic values of PLAUR in pan-cancer based on The Cancer Genome Atlas and Genotype-Tissue Expression databases. PLAUR was upregulated in multiple cancers and was significantly associated with poor overall survival and disease-free survival only in patients with KIRC. Subsequently, the PVT1/SNHG15-hsa-miR-532-3p axis was identified as the most potential upstream regulatory network of PLAUR in KIRC. In addition, PLAUR expression was closely associated with tumor-infiltrating immune cells, tumor immunity biomarkers, and immunomodulator expression. Furthermore, we constructed a multiple-gene risk prediction signature according to the PLAUR-related immunomodulators (PRIs). A prognostic nomogram was then developed to predict the 1-, 3-, and 5-year survival probabilities of individuals. In conclusion, our study identified the PVT1/SNHG15-hsa-miR-532-3p-PLAUR axis and a prognostic signature of PRIs, which could be a reference for future clinical research.

## Introduction

Renal cell carcinoma (RCC) is a common urinary malignancy originating from the epithelium of renal tubules, the incidence of which has recently increased. Approximately 76,080 new cases of RCC were diagnosed each year in the United States in 2021, of which 13,780 cases resulted in death (1). Kidney renal clear cell carcinoma (KIRC) is the most common subtype (representing approximately 80–90% of RCC), characterized by a high metastasis rate and resistance to radiotherapy and chemotherapy (2, 3). Approximately 25–30% of patients with KIRC are diagnosed with distant metastases, and the 5-year survival rate drops to approximately 10% (4). With the development of modern medicine, a multi-modal tumor strategy including surgical resection, molecular targeted therapy, and immunotherapy has dramatically optimized the clinical efficacy (5). Nevertheless, 30% of patients with localized KIRC inevitably developed local recurrence or tumor progression (2, 6). Although some promising biomarkers have been discovered, the underlying mechanism of recurrence and metastasis of KIRC is unclear (7). Therefore, new molecular-based research and effective therapies are urgently needed as they would be of great value for guiding the clinical management of patients with KIRC.

The urokinase-type plasminogen activator receptor (PLAUR, also known as CD87), a component of the urokinase-type plasminogen activator (PLAU) system, contains three structurally homologous domains and anchors on the cell surface through a glycosylated glycan-lipid (8, 9). PLAUR is involved in the pericellular network of interacting proteolytic systems and drives various malignancy-related processes, including angiogenesis, cell differentiation, proliferation, and migration (10–12). PLAUR overexpression has been reported in several hematologic and most solid malignancies, including acute lymphocytic leukemia, myeloma (13), breast cancer (14), non-small cell lung cancer (15), bladder cancer (16), and colon cancer (9). Additionally, Bhuvarahamurthy et al. revealed that PLAUR expression was upregulated in KIRC samples, and its expression appeared to increase with tumor grade or stage (17). PLAUR expression is mostly confined to the tumor tissue (11). PLAUR plays an important role in innate and adaptive immune responses (18). Rijneveld et al. reported that lymphocyte migration and macrophage and neutrophil infiltration were affected in infected tissues without PLAUR (19). Furthermore, PLAUR promotes activated T cell recruitment (18). Moreover, the composition of tumor-infiltrating immune cells may modulate tumor progression and determine outcomes (20). Therefore, it is clinically useful to investigate further the relationship between PLAUR expression and tumor immune infiltration in KIRC.

The regulatory mechanism of competing endogenous RNAs (ceRNAs) was presented for the first time by Salmena et al. in 2011 (21). This theory holds that ceRNA can competitively bind to microRNAs (miRNAs) and indirectly affect the gene silencing caused by miRNA. Long noncoding RNAs (lncRNAs) can adsorb onto miRNAs to regulate target gene expression (22). Currently, the ceRNA network has been shown to play a vital role in the occurrence and progression of multiple cancers (23). However, the significance of the key lncRNA–miRNA–PLAUR ceRNA network in KIRC needs further investigation.

In the present study, we first evaluated the differential expression and prognostic values of PLAUR in multiple cancers. Next, upstream miRNAs of PLAUR and upstream lncRNAs of candidate miRNAs were explored in KIRC. The associations between PLAUR and immune cell infiltration as well as biomarkers of immune cells were investigated using diverse authoritative databases. Further, we constructed a ceRNA (PVT1/SNHG15-hsa-miR-532-3p-PLAUR) regulatory network and a PLAUR-related immunomodulators (PRIs) signature to predict the prognosis of KIRC. The results of this study will enhance our understanding of the pathogenesis of KIRC and provide new molecular and therapeutic strategies for patients with KIRC.

## Materials and methods

### Data acquisition and pan-cancer PLAUR expression profile analysis

The mRNA expression profiles of 20 cancers were obtained from the University of California Santa Cruz Xena Browser (https://xenabrowser.net) (24). These transcriptomics data were normalized and analyzed using the “limma” R package. The differential expression levels of PLAUR in the different tumor and normal samples were analyzed using the Kruskal–Wallis test. The full list of 20 cancer types and their abbreviations is presented in **Table S1**.

### GEPIA database analysis

Based on The Cancer Genome Atlas (TCGA, https://portal.gdc.cancer.gov/) and Genotype-Tissue Expression (GTEx, https://gtexportal.org/) data, the Gene Expression Profiling Interactive Analysis (GEPIA, http://gepia.cancer-pku.cn/) is a free tool that delivers customizable functionalities, including differential expression and survival analyses (25). Additionally, we utilized the “Boxplots” module of GEPIA2 to analyze the expression profiles of PLAUR in diverse cancer types. Similarly, PLAUR-related lncRNA expression was also examined in KIRC. In addition, the “Survival Plots” module was applied to investigate overall survival (OS) and disease-free survival (DFS) data of the individual cancers in detail. Hazard ratios (HR) and *P* values were calculated using the log-rank test.

### StarBase database analysis

The miRNA–mRNA and lncRNA–miRNA interactions were predicted using the StarBase (http://starbase.sysu.edu.cn/) database (26). First, we used the StarBase database, which contained seven prediction algorithms (PITA, RNA22, miRmap, microT, miRanda, PicTar, and TargetScan), to predict the potential upstream binding miRNAs of PLAUR. Only the predicted miRNAs observed in one or more algorithms were regarded as potential miRNAs of PLAUR for the subsequent analysis. Furthermore, StarBase was employed to predict the potential lncRNAs that might bind to the above potential miRNAs. Furthermore, we explored the expression levels of miRNAs and lncRNAs in the KIRC samples and compared them with the controls. Moreover, the Kaplan–Meier survival analysis was used to assess the prognostic value of the potential targets. The expression correlation analysis for miRNA–mRNA, lncRNA–miRNA, and lncRNA–mRNA was further analyzed in the KIRC samples.

### Immune cell infiltration of PALUR in KIRC patients

CIBERSORT, a deconvolution algorithm developed by Newman (27), was employed to quantify the relative abundance of the 22 immune cell types in individual KIRC samples with the RNA-sequencing data from TCGA database. Only samples with CIBERSORT *P*< 0.05 were enrolled in this analysis. According to the median PLAUR expression, the KIRC samples were split into low- and high-expression groups. The infiltration levels of the immune cells were compared between the two groups and analyzed using the Wilcoxon rank-sum test. The correlation analysis among these infiltrating immune cells was performed using the Spearman test.

### TIMER database analysis

Tumor Immune Estimation Resource (TIMER, https://cistrome.shinyapps.io/timer/) is an integrative web interface used for the comprehensive analysis of tumor-infiltrating immune cells (28). We obtained six infiltrating immune cells (B cells, CD4+ T cells, CD8+ T cells, neutrophils, macrophages, and dendritic cells) in the KIRC samples using this dataset. The “somatic copy number alternation (SCNA)” module was used to compare the tumor infiltration levels under diverse SCNAs of PLAUR. The correlations between PLAUR expression and levels of immune cell infiltration were analyzed using TIMER.

### PLAUR-related immunomodulators

The immunophenoscore (IPS) was used to predict the immunotherapeutic responses by The Cancer Immunome Atlas (TCIA; https://tcia.at/), as described previously (29). TISIDB (http://cis.hku.hk/TISIDB) is one of the most comprehensive web portals for data on tumor and immune system interactions, comprising several types of data resources in onco-immunology (30). We utilized this database to retrieve the immunomodulators (immunopotentiators and immunosuppressants) associated with PLAUR. Immunomodulators closely correlated with the expression level of PLAUR were identified for further analysis (Spearman analysis, *P*< 0.05). The protein–protein interaction (PPI) networks of PLAUR-related immunomodulators (PRIs) were then generated using the STRING database (https://string-db.org/) (31). Gene Ontology (GO) and Kyoto Encyclopedia of Genes and Genomes (KEGG) pathway enrichment analyses were performed using the resulting protein network genes with the Metascape database (http://metascape.org) (32).

### Construction and validation of PRIs signature

Subsequently, we sought to construct a prognostic signature from the PRIs to assess the outcomes of patients with KIRC based on TCGA database. The differentially expressed PRIs (DEPRIs) between the 536 KIRC and normal samples were selected with a preset threshold of |log2 fold change (FC)| ≥ 2 and false discovery rate (FDR) < 0.05. The DEPRIs highly associated with OS were then determined as prognostic DEPRIs (*P*< 0.05) *via* univariate Cox regression. Further, the least absolute shrinkage and selection operator (Lasso) regression was applied to these prognostic DEPRIs to reduce the complexity of the model and control overfitting. We constructed the prognostic signature according to the expression levels and corresponding coefficients with the multivariate Cox regression. The risk score formula for each patient was as follows: risk score = $\overset{n}{\underset{i=1}{\sum }}\beta \left(i\right)\times x\left(i\right)$ , where *β*(*i*) and *x*(*i*) indicate the expression level and coefficient of gene, respectively. The Kaplan–Meier survival curve, the time-dependent receiver operating characteristic (ROC) curves and the area under the curve (AUC), principal component analysis (PCA), and t-distributed stochastic neighbor embedding (t-SNE) were employed to evaluate the performance of the constructed PRIs signature in TCGA cohort. E-MTAB-1980 with OS data downloaded from the EMBL-EBI database (https://www.ebi.ac.uk/) was used as an independent validation cohort.

### Development of nomogram

Independent prognostic factors were evaluated by Cox regression. In clinical research, nomograms are widely utilized as a quantitative tool to accurately assess cancer patients’ outcomes (33). In this study, a nomogram was developed to optimize the predictive performance for patients with KIRC by incorporating risk scores and the above independent prognostic factors. Afterward, the calibration and ROC curves were drawn, and decision curve analysis (DCA) was conducted to evaluate the accuracy, discrimination, and practicality of the nomogram, respectively.

### Statistical analysis

All statistical examinations in this study were performed using database-derived tools or the R language (34). Values of *P*< 0.05 and log-rank *P*< 0.05 were regarded as statistically significant.

## Results

### Expression profile of PLAUR in pan-cancer

**Figure 1:**
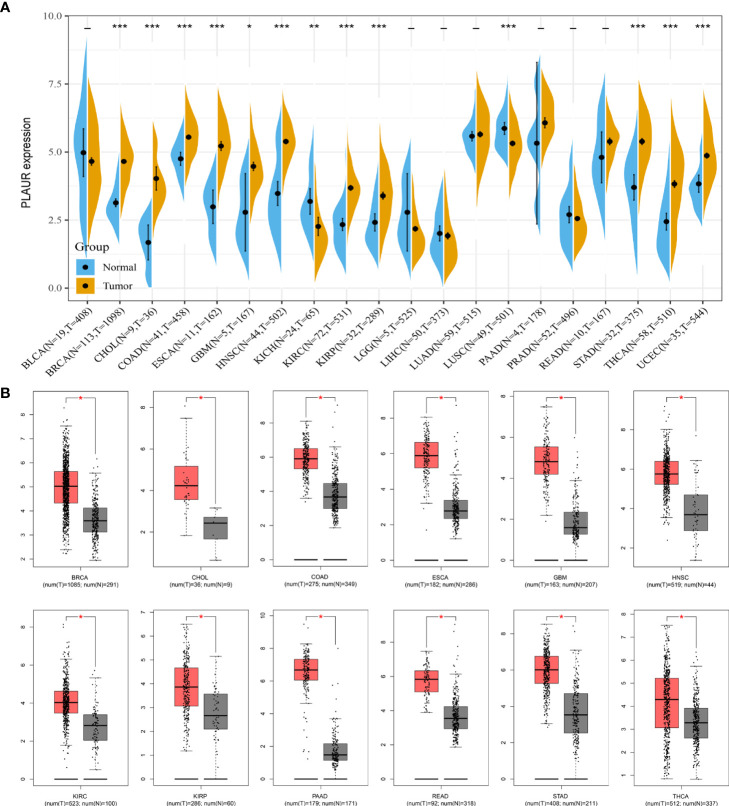
Expression analysis of PLAUR in pan-cancer. **(A)** The expression of PLAUR in 20 neoplastic tissues based on TCGA database. **(B)** The expression of PLAUR was higher in 12 cancer tissues compared with corresponding TCGA and GTEx normal tissues. *P value< 0.05; **P value< 0.01; ***P value < 0.001.

As the expression level of the PLAUR gene in pan-cancer has not yet been precisely determined, we utilized TCGA and GTEx databases for the differential expression analysis of PLAUR mRNA in the 20 most prevalent types of human cancer. Based on TCGA database, we discovered that PLAUR was significantly upregulated in 11 cancer types, including BRCA, CHOL, COAD, ESCA, GBM, HNSC, KIRC, KIRP, STAD, THCA, and UCEC, as compared to that in normal tissues; however, the PLAUR mRNA was downregulated in two cancers, i.e., KICH and LUSC (*P<* 0.05) (**Figure 1A**). No statistically significant difference was observed in the regulation of PLAUR in BLCA, LGG, LIHC, LUAD, PAAD, PRAD, and READ. Considering that TCGA database lacks normal tissue data for some cancers, we then utilized the GEPIA database (including TCGA and GTEx databases) to verify further the differences in the expression of PLAUR in these 20 cancers. On combining the RNA-sequencing data of the normal and tumor tissues from the GTEx and TCGA databases, PLAUR expression in tumor tissues of BRCA, CHOL, COAD, ESCA, GBM, HNSC, KIRC, KIRP, PAAD, READ, STAD, and THCA was found to be significantly higher than that in the corresponding control tissues (**Figure 1B**). Taken together, PLAUR may play a key regulatory role in the carcinogenesis of the 10 types of cancer, i.e., BRCA, CHOL, COAD, ESCA, GBM, HNSC, KIRC, KIRP, STAD, and THCA.

### Prognostic analysis of PLAUR in pan-cancer

**Figure 2:**
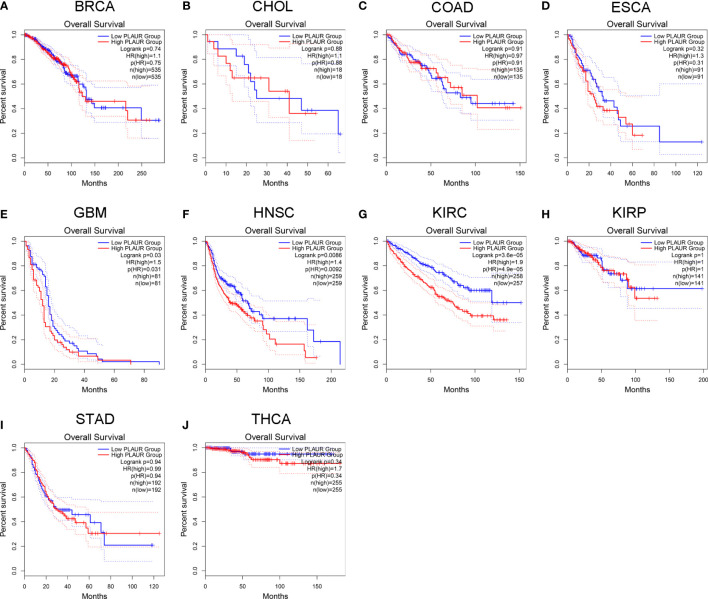
Kaplan-Meier survival curve for OS in 10 different tumor types using the GEPIA database. **(A–J)** BRCA, CHOL, COAD, ESCA, GBM, HNSC, KIRC, KIRP, STAD, THCA.

**Figure 3:**
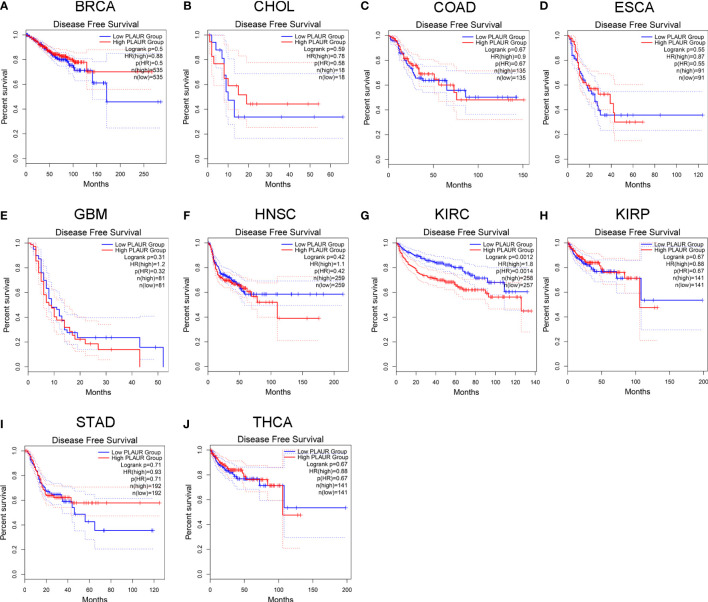
Kaplan-Meier survival curve for DFS in 10 different tumor types using the GEPIA database. **(A–J)** BRCA, CHOL, COAD, ESCA, GBM, HNSC, KIRC, KIRP, STAD, THCA.

To understand the association between PLAUR and tumor prognosis, Kaplan–Meier curves were constructed to evaluate the OS and DFS of the above 10 cancer types (BRCA, CHOL, COAD, ESCA, GBM, HNSC, KIRC, KIRP, STAD, and THCA) using the GEPIA database. The cancer samples were sorted into high and low PLAUR expression groups, using the median expression value of PLAUR. The OS curves showed that cases with higher PLAUR expression such as GBM, HNSC, and KIRC were associated with poor prognoses (**Figure 2**). Furthermore, the DFS curves suggested a correlation between high PLAUR expression and poor prognosis in the KIRC samples (**Figure 3**). The other cancers did not show any statistically significant difference indicating an association between PLAUR expression and survival-predicting ability. Taken together, these results revealed that PLAUR might serve as a biomarker for unfavorable outcomes in KIRC.

### Prediction of upstream miRNAs of PLAUR

**Figure 4:**
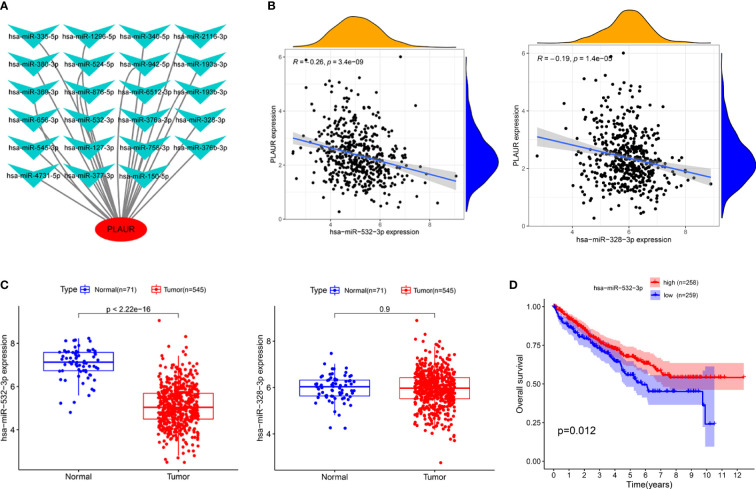
Identification of hsa-miR-532-3p as a potential upstream miRNA of PLAUR in KIRC using the StarBase database. **(A)** The miRNA-PLAUR regulatory network. **(B)** The expression correlation between hsa-miR-532-3p, hsa-miR-328-3p and PLAUR in KIRC. **(C)** The expression of hsa-miR-532-3p and hsa-miR-328-3p in KIRC tissues compared with normal controls. **(D)** The prognostic value of hsa-miR-532-3p in KIRC.

To determine whether PLAUR was regulated by certain noncoding RNAs (ncRNAs), we first investigated the upstream regulatory miRNAs that possibly target PLAUR and ultimately identified 23 miRNAs. An miRNA–RNA regulatory network, consisting of 23 miRNA–PLAUR relationships, was established and visualized using Cytoscape (**Figure 4A**). Based on the ceRNA hypothesis, the level of miRNA expression should be inversely correlated with that of mRNA expression (21). Therefore, the expression correlation between PLAUR and the 23 upstream regulatory miRNAs was further analyzed in patients with KIRC using the StarBase database (**Table 1**). We noticed two significant inverse correlation pairs, i.e., hsa-miR-328-3p-PALUR and hsa-miR-532-3p-PLAUR (all *P*< 0.05) (**Figure 4B**). No difference was found in the correlation of expression between PLAUR and the other 21 predicted miRNAs (**Table 1**). To further confirm whether hsa-miR-328-3p and hsa-miR-532-3p influenced KIRC, the expression levels of these two miRNAs in KIRC and normal tissues were analyzed. Significant differences in hsa-miR-532-3p were found in the KIRC samples compared to normal kidney tissues (**Figure 4C**), but not in hsa-miR-328-3p. Notably, combined with the OS analysis, hsa-miR-532-3p was significantly downregulated, and its downregulation was correlated with poor clinical outcomes in patients with KIRC (**Figure 4D**). The above results suggest that hsa-miR-532-3p may be the most potentially regulated miRNA of PLAUR in KIRC.

**Table 1:** The expression correlation analysis between predicted miRNAs and PLAUR and the differential expression analysis of predicted miRNAs.

Gene	miRNA	cor	pvalue	logFC	diffPval
PLAUR	hsa-miR-532-3p	-0.257	3.36E-09 ^a^	-1.98	1.66E-34 ^a^
PLAUR	hsa-miR-328-3p	-0.190	1.39E-05 ^a^	-0.024	9.02E-02
PLAUR	hsa-miR-1296-5p	-0.054	2.16E-01	-0.691	1.67E-13 ^a^
PLAUR	hsa-miR-524-5p	-0.054	2.21E-01	0.002	4.71E-01
PLAUR	hsa-miR-545-3p	-0.002	9.68E-01	0.022	5.66E-02
PLAUR	hsa-miR-6512-3p	0.003	9.44E-01	-0.069	3.07E-09 ^a^
PLAUR	hsa-miR-340-5p	0.042	3.37E-01	0.644	1.68E-07 ^a^
PLAUR	hsa-miR-876-5p	0.055	2.11E-01	-0.053	5.11E-06 ^a^
PLAUR	hsa-miR-4731-5p	0.059	1.79E-01	0.012	3.47E-02 ^a^
PLAUR	hsa-miR-127-3p	0.084	5.65E-02	-1.30	1.80E-16 ^a^
PLAUR	hsa-miR-380-3p	0.096	2.91E-02	-0.027	3.84E-02 ^a^
PLAUR	hsa-miR-2116-3p	0.127	3.84E-03 ^a^	0.088	1.23E-01
PLAUR	hsa-miR-758-3p	0.127	3.82E-03 ^a^	-0.622	6.67E-08 ^a^
PLAUR	hsa-miR-335-5p	0.141	1.33E-03 ^a^	-1.54	4.55E-24 ^a^
PLAUR	hsa-miR-656-3p	0.150	6.00E-04 ^a^	-0.097	3.46E-03 ^a^
PLAUR	hsa-miR-376a-3p	0.158	3.01E-04 ^a^	-0.096	3.83E-03 ^a^
PLAUR	hsa-miR-369-3p	0.174	7.15E-05 ^a^	-0.071	3.53E-01
PLAUR	hsa-miR-376b-3p	0.176	5.71E-05 ^a^	-0.256	1.02E-05 ^a^
PLAUR	hsa-miR-377-3p	0.194	8.66E-06 ^a^	-0.204	3.10E-05 ^a^
PLAUR	hsa-miR-942-5p	0.208	1.86E-06 ^a^	0.544	7.70E-10 ^a^
PLAUR	hsa-miR-150-5p	0.213	1.13E-06 ^a^	0.713	2.04E-06 ^a^
PLAUR	hsa-miR-193b-3p	0.263	1.37E-09 ^a^	-0.197	7.16E-02
PLAUR	hsa-miR-193a-3p	0.267	7.65E-10 ^a^	0.856	1.48E-17 ^a^

a These results are statistically significant.

### Prediction of upstream lncRNAs of hsa-miR-532-3p

**Figure 5:**
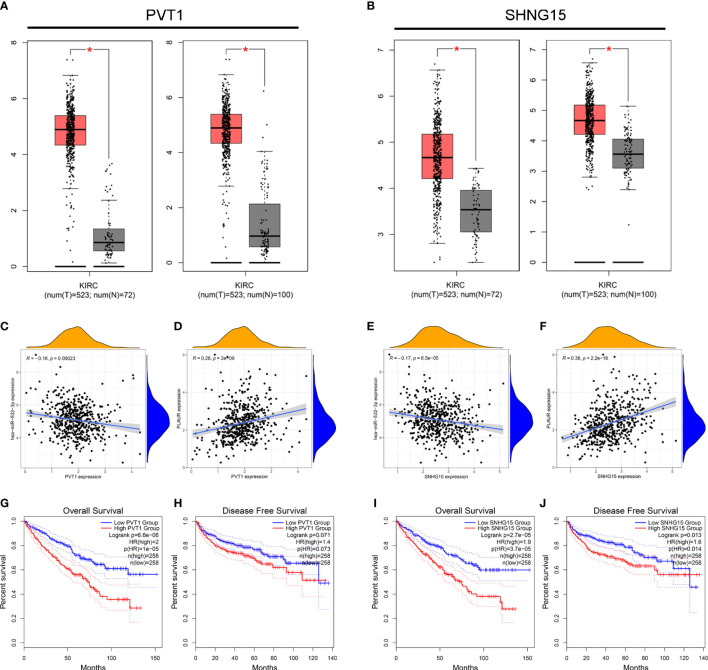
Identification of PVT1 and SNHG15 as two potential upstream lncRNAs of hsa-miR-532-3p-PLAUR axis in KIRC using the StarBase database. **(A–B)** The differential expression of PVT1 **(A)** and SNHG15 **(B)** in KIRC samples and “TCGA normal” or “TCGA and GTEx normal” samples. **(C-D)** The expression correlation between PVT1 and hsa-miR-532-3p **(C)**, PVT1 and PLAUR **(D)** in KIRC. **(E–F)** The expression correlation between SNHG15 and hsa-miR-532-3p **(E)**, SNHG15 and PLAUR **(F)** in KIRC. **(G–H)** Kaplan-Meier survival curve of OS for PVT1 **(G)** and SNHG15 **(H)**. **(I–J)** Kaplan-Meier survival curve of DFS for PVT1 **(G)** and SNHG15 **(H)** *p<0.05.

We used the StarBase database to identify the potential upstream lncRNAs that regulated hsa-miR-532-3p and determined 121 possible lncRNAs. Accordingly, we established hsa-miR-532-3p-lncRNA regulatory networks (**Table S2**). The differential expression levels of these lncRNAs in KIRC were then detected using GEPIA. Among all the 121 potential upstream lncRNAs, only PVT1 and SNHG15 expression levels were remarkably upregulated in the KIRC samples compared to those in the controls (**Figures 5A, B**). Afterward, we evaluated the prognostic values of PVT1 and SNHG15 in KIRC. The Kaplan–Meier survival analysis suggested high expression levels of PVT1 and SNHG15, indicating poor OS and DFS in patients with KIRC (**Figure 5C–F**). Based on the theory proposed by Salmena et al., lncRNAs might act as ceRNAs by sponging miRNA to regulate mRNA expression (21). Thus, lncRNA expression should be positively correlated with mRNA expression and negatively correlated with miRNA expression. Further, we investigated the correlation between the expression levels of two lncRNAs (PVT1 and SNHG15) and hsa-miR-532-3p or PLAUR in KIRC (**Figures 5G–J**). The result was consistent with the theoretical prediction of Salmena et al. According to the differential expression, survival, and correlation analyses, PVT1 and SNHG15 were predicted to be the most potential upstream lncRNAs of the hsa-miR-532-3p/PLAUR axis in KIRC.

### The relationship between PLAUR Expression and immune cell infiltration

The expression spectrums and the relative proportions of the 22 immune cells in KIRC were explored and assessed through the CIBERSORT algorithm to investigate the infiltrations of specific immune cell subpopulations. Then, we constructed a bar plot to display the landscapes of different cell subtypes in each sample after screening the samples with *P* ≥ 0.05 (**Figure S1A**). The correlation analyses of the 22 cell subtypes showed weak-to-moderate correlation, and the strongest positive correlations were found between memory-resting CD8+ and CD4+ T cells (**Figure S1B**). Additionally, compared with the PLAUR low expression group, the fractions of plasma cells, memory-activated CD4 T cells, regulatory T cells (Tregs), M0 macrophages, activated mast cells, and neutrophils were significantly higher, whereas those of memory-resting CD4+ T cells, resting NK cells, monocytes, macrophages M1, and resting mast cells were significantly lower in the PLAUR high expression group (**Figure S1C**). These findings indicated that the activation of some immune cells appeared to reflect the impact of PLAUR on the immune system.

**Figure 6:**
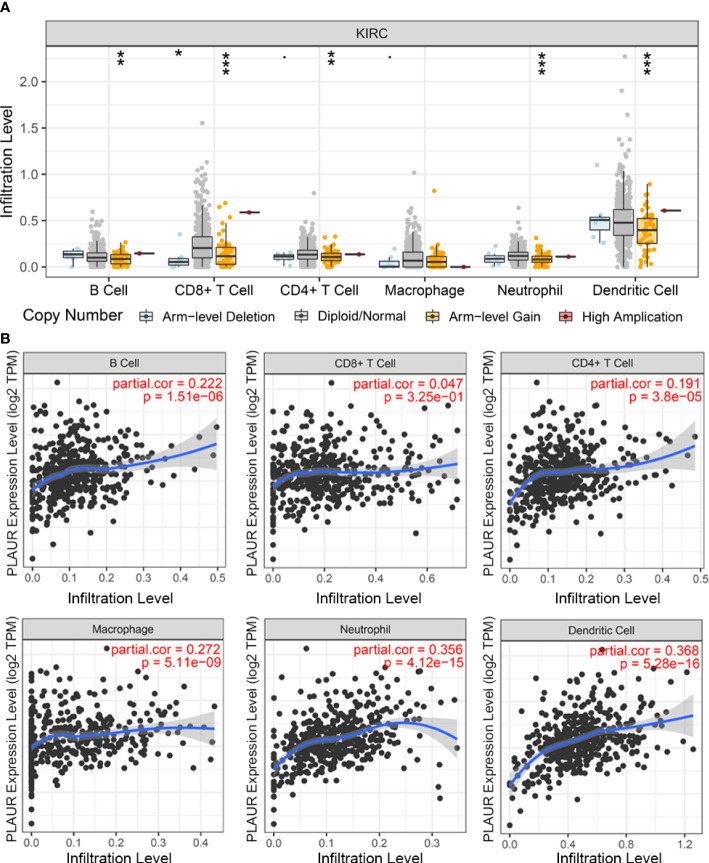
The relationship between expression of PLAUR and immune cell infiltration in KIRC. **(A)** Association between PLAUR gene copy number and immune cell infiltration levels in KIRC. **(B)** The relationship of PLAUR expression with six various infiltrating immune cells in KIRC. *p<0.05;**p<0.01; ***p<0.001.

The PLAUR gene is a key gene involved in coagulation and fibrinolysis, which are under complex regulation by inflammation and the local recruitment of leukocytes in the tumor microenvironment (35, 36). Hence, using the TIMER database, we further explored the underlying relationships between the SCNA of PLAUR and six different infiltrating immune cells. Besides macrophages, the copy numbers of PLAUR varied significantly in all the infiltrating immune cells, including B cells, CD8+ T cells, CD4+ T cells, neutrophils, and dendritic cells in the KIRC samples (**Figure 6A**). Moreover, we found that samples with arm-level deletion by PLAUR showed lower levels of immune infiltrates than diploid/normal samples, indicating the effect of arm-level deletion on the infiltration level of immune cells in KIRC. A correlation analysis provided more insights into the mechanism of PLAUR in KIRC. The correlation analysis of PLAUR expression with infiltrating immune cells demonstrated that PLAUR expression was positively correlated with the infiltration of all immune cells except CD8+ T cells (**Figures 6B**). To further clarify the influence of PLAUR expression on tumor immune cells, we conducted an expression correlation analysis between PLAUR expression and the biomarkers released by various infiltrating immune cells. PLAUR expression was significantly linked to most (20/22) biomarkers (**Table 2**). Except NOS2 (r< 0), all the 19 biomarkers of infiltrating immune cells presented positive correlations with PLAUR expression. This confirmed the PLAUR expression was positively linked to immune cell infiltration.

**Table 2:** Correlation analysis between PLAUR and biomarkers of immune cells in KIRC.

Immune cell	Biomarker	cor	pvalue
B cell	CD19	0.377	0 ^a^
B cell	CD79A	0.368	0 ^a^
CD8+ T cell	CD8A	0.217	4.36E-07 ^a^
CD8+ T cell	CD8B	0.215	5.84E-07 ^a^
CD4+ T cell	CD4	0.442	0 ^a^
M1 macrophage	NOS2	-0.132	2.27E-03 ^a^
M1 macrophage	IRF5	0.193	7.57E-06 ^a^
M1 macrophage	PTGS2	0.288	1.41E-11 ^a^
M2 macrophage	CD163	0.329	7.31E-15 ^a^
M2 macrophage	VSIG4	0.440	0 ^a^
M2 macrophage	MS4A4A	0.399	0 ^a^
Neutrophil	CEACAM8	-0.026	5.52E-01
Neutrophil	ITGAM	0.371	0 ^a^
Neutrophil	CCR7	0.370	0 ^a^
Dendritic cell	HLA-DPB1	0.311	2.31E-13 ^a^
Dendritic cell	HLA-DQB1	0.199	3.71E-06 ^a^
Dendritic cell	HLA-DRA	0.308	4.43E-13 ^a^
Dendritic cell	HLA-DPA1	0.240	2.02E-08 ^a^
Dendritic cell	CD1C	0.120	5.45E-03 ^a^
Dendritic cell	NRP1	-0.058	1.80E-01
Dendritic cell	ITGAX	0.432	0 ^a^

a These results are statistically significant.

### Association analysis between PLAUR and immunomodulators

We aimed to investigate whether PLAUR was related to immunomodulators, as the introduction of immunotherapy has considerably transformed the cancer treatment landscape. To explore the association with response to immunotherapy, we investigated the association between PLAUR-high vs PLAUR-low expression groups in some established immune-related signatures introduced by Braun et al. (37). The signature analysis was performed using four immune-related signatures listed in **Table S3**. There was a significant difference in myeloid cell infiltration (IMmotion150 Myeloid) (38) between the high- and low- PLAUR expression groups for either the Anti-PD1 treatment or the mTOR treatment (**Figures S2A, S2B**). No significant difference was detected in IMmotion150 Angio (38), T effector cell infiltration (IMmotion150 Teff) (38), and Javelin (39).

In addition, on investigating the significance of PLAUR to assess the effect of immunotherapy using TCIA, the results illustrated that the relative probabilities of responding to CTLA4-positive/PDL1-negative treatments in the low PLAUR group were higher than those in the high PLAUR group (**Figures S2C–S2E**).

**Figure 7:**
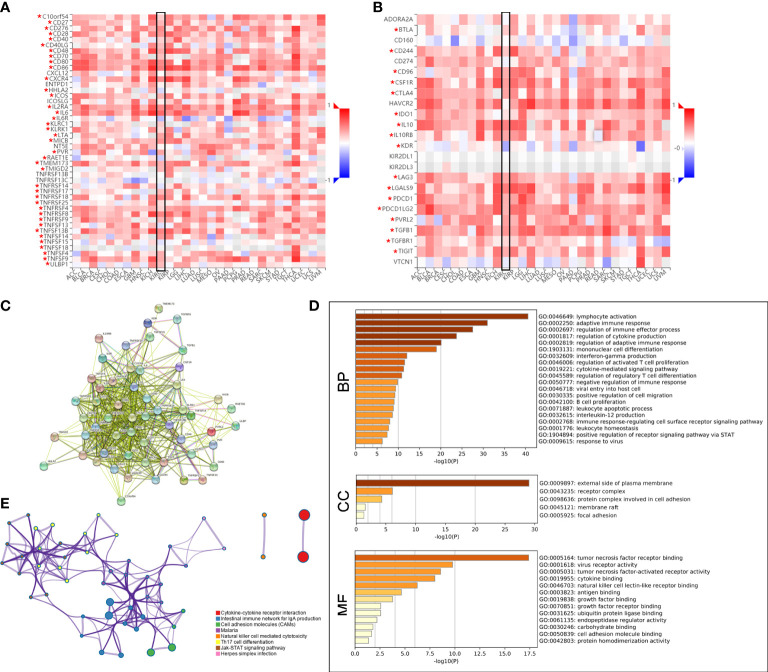
Identification of PLAUR-related immunomodulators (PRIs). **(A–B)** The correlation heatmaps of PLAUR with immunopotentiators **(A)** and immunosuppressants **(B)**. **(C)** Protein–protein network of 55 PRIs. **(D)** Gene Ontology annotation of 55 PRIs. **(E)** Kyoto Encyclopedia of Genes and Genomes pathway analysis of 55 PRIs. *P value< 0.05.

Subsequently, the Spearman correlations between PLAUR expression and immunopotentiators as well as immunosuppressants were analyzed using the TISIDB database. A total of 38 immunopotentiators (**Figure 7A**) and 17 immunosuppressants (**Figure 7B**) were identified to be significantly correlated with PLAUR expression in KIRC (*P*< 0.05). The PPI of these 55 PRIs is presented in **Figure 7C**. As per the Metascape database, the most abundant biological process related to the 55 PRIs were immune events, particularly lymphocyte activation (**Figure 7D**). In terms of cellular component and molecular function analyses, these PRIs were mainly enriched in the external side of the plasma membrane and tumor necrosis factor receptor binding, respectively (**Figure 7D**). The result from the KEGG analysis revealed that the intestinal immune networks for IgA production, natural killer cell-mediated cytotoxicity, cell adhesion molecules, and Jak-STAT signaling pathways were associated with PLAUR-mediated immune system processes (**Figure 7E**).

### Construction and validation of the PRIs signature for KIRC

**Figure 8:**
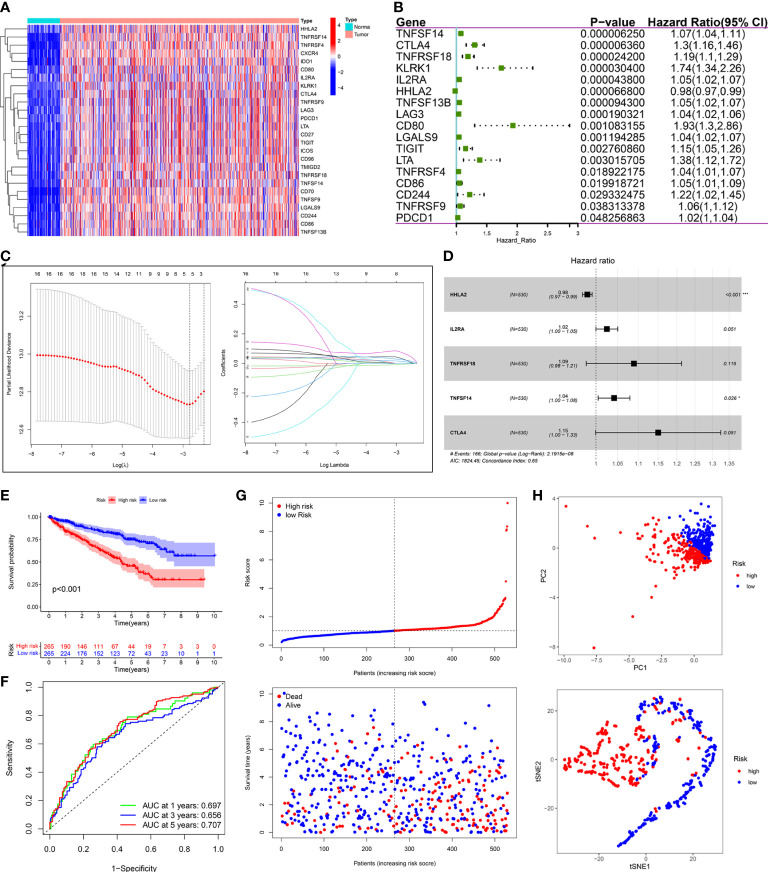
Construction of prognostic PRIs signature based on the TCGA KIRC cohort. **(A)** The heatmaps of 26 differentially expressed PRIs in KIRC tissues and normal tissues. **(B)** Forest plot of univariate Cox regression analysis of PRIs related to the OS. **(C)** LASSO coefficients profiles of the 5 PRIs in KIRC. **(D)** Forest plot of multivariate Cox regression analysis of 5 PRIs in KIRC. **(E)** Kaplan-Meier analysis of KIRC patients stratified by the median risk score in the TCGA cohort. **(F)** The time-dependent ROC curve for OS in the TCGA cohort. **(G)** The distribution of the risk score and OS status in the TCGA cohort. **(H)** PCA and t-SNE analyses in the TCGA cohort.

Compared with normal samples, 26 out of 55 PRIs were differentially expressed in tumor samples using the screening criteria (|log2FC|>2 and FDR<0.05). A heatmap was generated to illustrate the expression profiles of these 26 DEPRIs in KIRC (**Figure 8A**). Among them, only one gene, HHLA2, was downregulated in the KIRC tissues, whereas the remaining 25 genes were upregulated. The univariate Cox regression analysis was employed to investigate the prognostic values of these 26 DEPRIs in KIRC in TCGA database, and 17 DEPRIs were found to be closely related to OS (*P*< 0.05) (**Figure 8B**). The 17 candidate genes were further studied using LASSO (**Figure 8C**) and a multivariate (**Figure 8D**) Cox regression analysis. Ultimately, five target genes were retained to construct the prognostic PRIs signature. Then, the coefficient values were extracted to calculate the risk score for each patient with the following formula: risk score = (-0.0196 × the expression value of HHLA2) + (0.0245× the expression value of IL2RA) + (0.0855× the expression value of TNFRSF18) + (0.0407× the expression value of TNFSF14) + (0.1404× the expression value of CTLA4).

**Figure 9:**
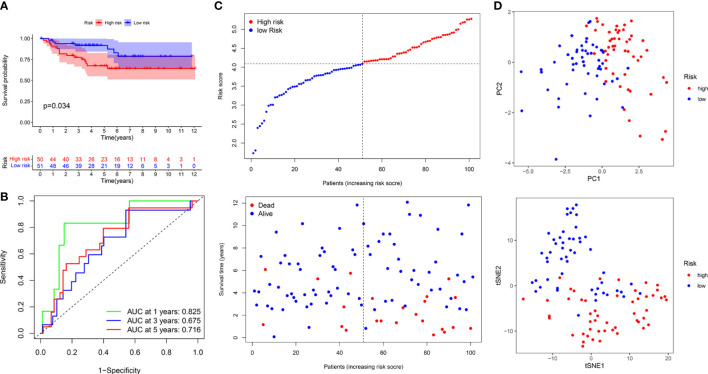
The prognostic value of PRIs prognostic for KIRC patients in an independent E-MTAB-1980 validation cohort. **(A)** Kaplan-Meier analysis of KIRC patients stratified by the median risk score in the E-MTAB-1980 cohort. **(B)** The time-dependent ROC curve for OS in the E-MTAB-1980 cohort. **(C)** The distribution of the risk score and OS status in the E-MTAB-1980 cohort. **(D)** PCA and t-SNE analyses in the E-MTAB-1980 cohort.

The median risk score was used to classify patients into the low- and high-risk groups. The Kaplan–Meier curve showed that patients of the high-risk group suffered a poor prognosis compared to those of the low-risk group (**Figure 8E**). Time-dependent ROC curves showed that the PRIs signature harbored a satisfactory performance to predict OS in patients with KIRC (1-, 3-, and 5-year AUC was 0.697, 0.656, and 0.707, respectively) (**Figure 8F**). The scatterplot of the risk score and survival status showed that the mortality rate of patients increased with the increase in risk score (**Figure 8G**). Moreover, PCA and t-SNE analyses were performed to confirm the diverse directions between the two risk groups (**Figure 8H**). We performed the same analyses in an independent validation cohort to determine whether the PRIs have prognostic significance in other populations. Analogous results were achieved in the E-MTAB-1980 cohort (**Figures 9A–D**). The above results reflected that the PRIs signature might precisely and steadily predict survival outcomes in patients with KIRC.

### Independent prognostic value of the PRIs signature

We performed univariate and multivariate Cox regression analyses to investigate whether the PRIs signature was a clinically independent prognostic factor for KIRC. The univariate Cox regression analysis showed that the signature-based risk score was closely related to the OS of patients with KIRC (HR = 1.282, *P<* 0.001) in TCGA database (**Figure S3A**). In contrast, the multivariate Cox analysis revealed that the signature could work as an independent prognostic factor (HR = 1.196, *P* = 0.002) (**Figure S3B**). These results were validated in the E-MTAB-1980 cohort (**Figure S3C, S3D**).

### Development of the nomogram and the evaluation of predictive effectiveness

**Figure 10:**
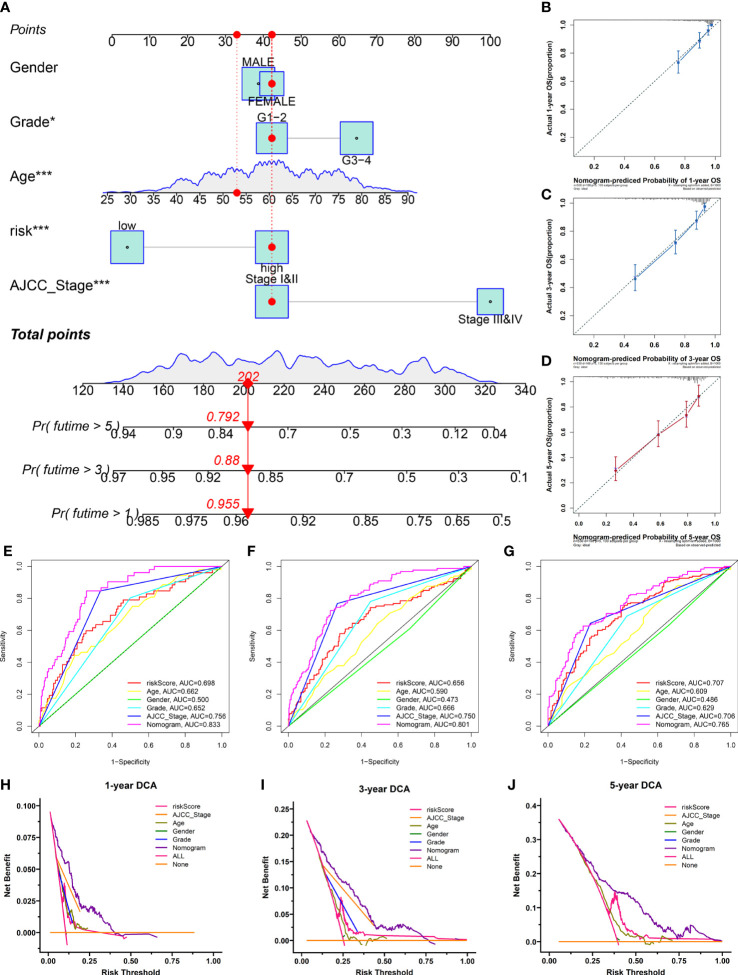
The nomogram was developed for prognostic probabilities prediction in the TCGA database. **(A)** Development of a nomogram for predicting survival probability of KIRC patients at 1-, 3-, and 5-years. **(B–D)** The calibration curves of the nomogram at 1-, 3-, and 5-years. **(E–G)** The time‐dependent ROC curves of the nomogram at 1-, 3-, and 5-years. **(H–J)** The DCA curves of the nomogram at 1-, 3-, and 5-years. *p<0.05;***p<0.001.

To provide clinicians with a quantitative approach to assessing the individual survival probability in patients with KIRC, we developed a nomogram incorporating the risk score and clinical features (including age, gender, grade, and AJCC stage) in TCGA cohort (**Figure 10A**). The calibration curves of the nomogram exhibited good concordance between the predicted and actual outcomes, revealing that the nomogram possessed better predictive ability (**Figures 10B–D**). The AUC values predicting the 1-, 3-, and 5-year OS of the nomogram were 0.833, 0.801, and 0.760, respectively, which outperformed all clinicopathological features (**Figures 10E–G**). The DCA suggested that the nomogram provided a higher overall net benefit than other clinical features for most threshold probabilities, demonstrating the potential clinical usefulness of the predictive nomogram (**Figures 10H–J**). Altogether, the nomogram might increase the predicted probability and confer some net benefit, enabling individualized prognosis prediction and helping clinical practice.

## Discussion

KIRC is one of the most lethal cancers, usually showing malignant progression and a high tumor recurrence rate (6). Although various therapeutic strategies are available for KIRC, patient survival rates remain poor (2, 5) Elucidating the molecular mechanism underlying KIRC pathogenesis and seeking potential biomarkers are critical for identifying novel therapeutic targets and improving the prognosis of patients with KIRC. PLAUR plays a crucial role in the initiation and progression of various cancers, including KIRC (11). However, the function and mechanism of PLAUR in patients with KIRC are still unclear and require further investigation.

In this study, we first analyzed the expression of PLAUR in pan-cancer using data from TCGA database, after which we used the GEPIA database to confirm the expression and prognostic potential of PLAUR. The survival analysis for PLAUR across 20 human cancers demonstrated that high PLAUR expression was significantly correlated with poor OS and DFS only in patients with KIRC. Two previous studies indicated that PLAUR expression was upregulated in KIRC tissues compared to normal tissues, and the expression appeared to increase with tumor grade or stage (17, 40). Concomitant to these reports, our results revealed the tumorigenic effect of PLAUR in KIRC.

LncRNA can interact with miRNA to participate in the regulation of target gene expression based on the ceRNA hypothesis (21, 41). Through the StarBase database, we predicted that upstream regulatory miRNAs potentially bind to PLAUR and determined 23 candidate miRNAs. Most of these miRNAs might serve as tumor suppressors in KIRC. For example, miR-127-3p inhibited the proliferation and metastasis of KIRC by targeting the CDKN3/E2F1 axis (42), and miR-335-5p suppressed KIRC cell proliferation and invasion by repressing BCL-W expression  (43). After a series of analyses for these 23 miRNAs, miR-532-3p was identified as the most potential miRNA of PLAUR, which was negatively associated with PLAUR expression; it was overexpressed and predicted a bad outcome in KIRC. Moreover, Han et al. suggested that miR-532-3p inhibited the malignant progression of KIRC by downregulating the expression of ETS1, an oncogene associated with unfavorable prognosis in KIRC (44).

Subsequently, 121 upstream potential lncRNAs of the miR-532-3p-PLAUR axis were discovered. Two of the most potentially upregulated lncRNAs (PVT1 and SNHG15) were then determined by a series analysis. Emerging research has revealed that PVT1 and SNHG15 play important roles in the progression of various cancers, including KIRC. Yang et al. reported that PVT1 could serve as ceRNA in the context of KIRC and promoted cancer proliferation and migration *in vitro* and *in vivo* experiments (45). In addition, another study confirmed that the upregulated expression of SNHG15 induced the epithelial-mesenchymal transition process and accelerated the invasion and migration of KIRC by regulating the nuclear factor κB signaling pathway (46). Based on the above results, the PVT1/SNHG15-hsa-miR-532-3p-PLAUR axes were considered potential regulatory pathways in KIRC.

The infiltration of immune cells in the TME has previously been associated with the prognosis and immunotherapy outcomes of various human tumors, especially in patients with KIRC. The present study showed a significant positive correlation of PLAUR expression with various immune cells in KIRC. The activation of dendritic cells and CD8+ T cells was correlated with favorable prognoses in the majority of solid tumors, but with the poor prognosis of KIRC (47), which indicated that KIRC possessed a unique TME. Additionally, infiltrating CD4+ T cells and macrophages enhanced KIRC cell proliferation and invasion, respectively (48). PLAUR expression was also significantly positively correlated with the biomarkers of various infiltrating immune cells. These results suggest that tumor immune infiltration may influence PLAUR-mediated KIRC progression.

IMmotion150 is the first randomized study to evaluate the clinical activity of a combination of antiangiogenic agents and immune checkpoint inhibitors in untreated patients with mRCC (38). In this study, we observed that the relative probabilities of responding to ICI in the low PLAUR group were higher than those in the high PLAUR group using the TCIA database. Another interesting finding was the high PLAUR cases are enriched in the IMmotion150 myeloid signature, which has been previously associated with resistance to single-agent atezolizumab in the IMmotion150 trial (38, 49). Taken together, this data corroborate previous findings from the IMmotion150 trial and add further insights into the possible mechanisms of resistance to ICI in tumors with high myeloid inflammation.

The expression of immunomodulators substantially affects cancer treatment. Therefore, we further examined the relationship between PLAUR and immunomodulators (including 45 immunopotentiators and 24 immunosuppressants) and identified 55 PRIs using the TISIDB database. We performed functional enrichment analyses to investigate further the underlying biological processes and pathways associated with PLAUR in KIRC. A GO enrichment analysis suggested that the 55 PRIs were mainly involved in multiple immune responses. A KEGG pathway analysis of these PRIs showed that the Jak-STAT signaling pathway and cell adhesion molecules might be associated with PLAUR-mediated immune response. PLAUR blockade was reported to inhibit nasopharyngeal carcinoma cell migration and invasion by affecting the expression of phosphorylating Jak1 and STAT1 (50). Unsurprisingly, the Jak-STAT signaling pathway participates in almost all immunomodulatory processes, such as immune surveillance, inflammation, and tumor-driven immune escape (51). In addition, PLAUR could mediate cell adhesion and initiate intracellular signal transduction pathways (52). Cell adhesion molecules are important for cell-based tissue integrity and immune responses (53).

In this study, we also successfully constructed a PRIs signature to evaluate the prognosis of patients with KIRC. The patients were classified as low- and high-risk based on the median risk score. The Kaplan–Meier curve showed a great difference between the risk groups, and patients of the high-risk group had an unfavorable prognosis compared to those of the low-risk group. The time-dependent ROC curves, scatterplots of the risk score, and PCA analyses confirmed the favorable performance of the signature. Analogous results were achieved in an independent validation cohort. In addition, five PRIs (HHLA2, IL2RA, TNFRSF18, TNFSF14, and CTLA4) included in the signature were regarded as potential PLAUR-related biomarkers in KIRC, which were studied in other cancers including KIRC based on the current literature (7, 54–57). Finally, we developed a nomogram for personalized prognosis prediction by combining signature-based risk scores with various clinical features. These results demonstrated that the PRIs signature has significant prognostic implications in patients with KIRC.

**Figure 11:**
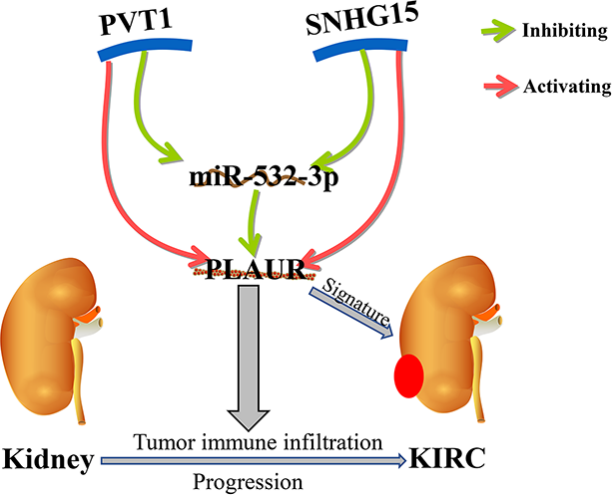
The model of PVT1/SNHG15-hsa-miR-532-3p-PLAUR axis in carcinogenesis of KIRC.

The main strengths of our study are that we have identified the PVT1/SNHG15-hsa-miR-532-3p-PLAUR axis for the first time and constructed a prognostic PRIs signature for patients with KIRC (**Figure 11**). However, this study does have certain limitations. First, the function and mechanism of the PVT1/SNHG15-hsa-miR-532-3p-PLAUR axis should be further studied experimentally. Second, the clinical application of the PLAUR axis needs to be validated and verified in clinical practice. Third, an independent study with larger sample size is warranted to validate the predictive power of the PRIs signature.

## Conclusion

High PLAUR expression was frequently observed in most common cancers and was significantly related to unfavorable outcomes (including OS and DFS) in KIRC. We constructed a PLAUR-related ceRNA regulatory network in KIRC, namely the PVT1/SNHG15-hsa-miR-532-3p-PLAUR axis. In addition, the current study suggested that PLAUR might exert its tumorigenic effect by regulating tumor immune cell infiltration and immunomodulatory expression. The risk signatures derived from PRIs were independently predictive of OS for patients with KIRC and have potentially substantial clinical significance.

## Data availability statement

Publicly available datasets were analyzed in this study. This data can be found here: UCSC Xena Browser (https://xenabrowser.net), and EMBL-EBI database (https://www.ebi.ac.uk/).

## Author contributions

YW and ZS designed the study and wrote this manuscript. SL organized the database. XZ and CX performed the statistical analysis. TL and JW revised the manuscript. All authors have seen and approved the final version of the manuscript. All authors contributed to the article and approved the submitted version.

## Funding

This work was supported by Natural Science Foundation of Guangdong Province (2018A030313261).

## Conflict of interest

The authors declare that the research was conducted in the absence of any commercial or financial relationships that could be construed as a potential conflict of interest.

## Publisher’s note

All claims expressed in this article are solely those of the authors and do not necessarily represent those of their affiliated organizations, or those of the publisher, the editors and the reviewers. Any product that may be evaluated in this article, or claim that may be made by its manufacturer, is not guaranteed or endorsed by the publisher.
