# Supplementary material for: Identification of PLAUR-related ceRNA and immune prognostic signature for kidney renal clear cell carcinoma
Source: Front Oncol. 2022 Aug 16;12:834524. doi: 10.3389/fonc.2022.834524 (PMC9424644; doi:10.3389/fonc.2022.834524)
Supplement: Supplementary file 1 [file DataSheet_1.docx]

Table S1. TCGA Study Abbreviations in manuscript.

| **Tumor Name** | **Abbreviations** |
| --- | --- |
| Bladder urothelial carcinoma | BLCA |
| Breast invasive carcinoma | BRCA |
| Cholangiocarcinoma | CHOL |
| Colon adenocarcinoma | COAD |
| Esophageal carcinoma | ESCA |
| Glioblastoma multiforme | GBM |
| Head and neck squamous cell carcinoma | HNSC |
| Kidney chromophobe | KICH |
| Kidney renal clear cell carcinoma | KIRC |
| Kidney renal papillary cell carcinoma | KIRP |
| Low grade glioma | LGG |
| Liver hepatocellular carcinoma | LIHC |
| Lung adenocarcinoma | LUAD |
| Lung squamous cell carcinoma | LUSC |
| Pancreatic adenocarcinoma | PAAD |
| Prostate adenocarcinoma | PRAD |
| Rectum adenocarcinoma | READ |
| Stomach adenocarcinoma | STAD |
| Thyroid carcinoma | THCA |
| Uterine corpus endometrial carcinoma | UCEC |

Table S2. The lncRNA/hsa-miR-532-3p regulatory network.

| **No.** | **miRNA** | **lncRNA** |
| --- | --- | --- |
| 1 | hsa-miR-532-3p | AL391244.1 |
| 2 | hsa-miR-532-3p | AL512413.1 |
| 3 | hsa-miR-532-3p | LINC01134 |
| 4 | hsa-miR-532-3p | MIR34AHG |
| 5 | hsa-miR-532-3p | CCDC18-AS1 |
| 6 | hsa-miR-532-3p | SLC16A1-AS1 |
| 7 | hsa-miR-532-3p | AL365436.2 |
| 8 | hsa-miR-532-3p | AC244034.3 |
| 9 | hsa-miR-532-3p | DNAJC27-AS1 |
| 10 | hsa-miR-532-3p | AC073046.1 |
| 11 | hsa-miR-532-3p | TBC1D8-AS1 |
| 12 | hsa-miR-532-3p | LINC01806 |
| 13 | hsa-miR-532-3p | AC016717.2 |
| 14 | hsa-miR-532-3p | AC010148.1 |
| 15 | hsa-miR-532-3p | AC104667.2 |
| 16 | hsa-miR-532-3p | AC012485.1 |
| 17 | hsa-miR-532-3p | AC006058.3 |
| 18 | hsa-miR-532-3p | PTPRG-AS1 |
| 19 | hsa-miR-532-3p | MBNL1-AS1 |
| 20 | hsa-miR-532-3p | LINC02015 |
| 21 | hsa-miR-532-3p | AC022498.2 |
| 22 | hsa-miR-532-3p | CTBP1-AS2 |
| 23 | hsa-miR-532-3p | NOP14-AS1 |
| 24 | hsa-miR-532-3p | AC025741.1 |
| 25 | hsa-miR-532-3p | HHIP-AS1 |
| 26 | hsa-miR-532-3p | AC098864.1 |
| 27 | hsa-miR-532-3p | AC012640.2 |
| 28 | hsa-miR-532-3p | AC113382.1 |
| 29 | hsa-miR-532-3p | AC034243.1 |
| 30 | hsa-miR-532-3p | AC021078.1 |
| 31 | hsa-miR-532-3p | AC011369.2 |
| 32 | hsa-miR-532-3p | HCG11 |
| 33 | hsa-miR-532-3p | AL080317.1 |
| 34 | hsa-miR-532-3p | AC018647.2 |
| 35 | hsa-miR-532-3p | AC007349.2 |
| 36 | hsa-miR-532-3p | AC005537.1 |
| 37 | hsa-miR-532-3p | SNHG15 |
| 38 | hsa-miR-532-3p | AC005072.1 |
| 39 | hsa-miR-532-3p | AC006333.2 |
| 40 | hsa-miR-532-3p | AF131215.2 |
| 41 | hsa-miR-532-3p | LZTS1-AS1 |
| 42 | hsa-miR-532-3p | AC055854.1 |
| 43 | hsa-miR-532-3p | AC107959.2 |
| 44 | hsa-miR-532-3p | EXTL3-AS1 |
| 45 | hsa-miR-532-3p | AC087623.3 |
| 46 | hsa-miR-532-3p | GASAL1 |
| 47 | hsa-miR-532-3p | AC027031.2 |
| 48 | hsa-miR-532-3p | PVT1 |
| 49 | hsa-miR-532-3p | AC107375.1 |
| 50 | hsa-miR-532-3p | BX255923.3 |
| 51 | hsa-miR-532-3p | MIR600HG |
| 52 | hsa-miR-532-3p | AL162586.1 |
| 53 | hsa-miR-532-3p | AL359091.3 |
| 54 | hsa-miR-532-3p | LINC00963 |
| 55 | hsa-miR-532-3p | PPP1R26-AS1 |
| 56 | hsa-miR-532-3p | C9orf139 |
| 57 | hsa-miR-532-3p | CASC2 |
| 58 | hsa-miR-532-3p | AP006621.5 |
| 59 | hsa-miR-532-3p | H19 |
| 60 | hsa-miR-532-3p | KCNQ1OT1 |
| 61 | hsa-miR-532-3p | AL133330.1 |
| 62 | hsa-miR-532-3p | AP006333.1 |
| 63 | hsa-miR-532-3p | NEAT1 |
| 64 | hsa-miR-532-3p | MALAT1 |
| 65 | hsa-miR-532-3p | AP002761.3 |
| 66 | hsa-miR-532-3p | RAB30-AS1 |
| 67 | hsa-miR-532-3p | U47924.1 |
| 68 | hsa-miR-532-3p | AC010168.2 |
| 69 | hsa-miR-532-3p | AC121338.2 |
| 70 | hsa-miR-532-3p | AC008147.2 |
| 71 | hsa-miR-532-3p | AC012531.2 |
| 72 | hsa-miR-532-3p | AC027290.2 |
| 73 | hsa-miR-532-3p | AC026362.2 |
| 74 | hsa-miR-532-3p | AL161772.1 |
| 75 | hsa-miR-532-3p | LINC01070 |
| 76 | hsa-miR-532-3p | AL135999.1 |
| 77 | hsa-miR-532-3p | AC005993.1 |
| 78 | hsa-miR-532-3p | AL356017.1 |
| 79 | hsa-miR-532-3p | SNHG10 |
| 80 | hsa-miR-532-3p | AL583810.1 |
| 81 | hsa-miR-532-3p | EIF3J-AS1 |
| 82 | hsa-miR-532-3p | AC090970.3 |
| 83 | hsa-miR-532-3p | TPM1-AS |
| 84 | hsa-miR-532-3p | IQCH-AS1 |
| 85 | hsa-miR-532-3p | LINC00052 |
| 86 | hsa-miR-532-3p | AC013489.1 |
| 87 | hsa-miR-532-3p | AC022167.2 |
| 88 | hsa-miR-532-3p | AC120114.4 |
| 89 | hsa-miR-532-3p | AC026471.6 |
| 90 | hsa-miR-532-3p | AC040162.3 |
| 91 | hsa-miR-532-3p | AC009078.3 |
| 92 | hsa-miR-532-3p | AC025287.3 |
| 93 | hsa-miR-532-3p | AC092127.1 |
| 94 | hsa-miR-532-3p | AC092384.3 |
| 95 | hsa-miR-532-3p | VPS9D1-AS1 |
| 96 | hsa-miR-532-3p | AC067852.2 |
| 97 | hsa-miR-532-3p | AC004477.1 |
| 98 | hsa-miR-532-3p | AC005839.1 |
| 99 | hsa-miR-532-3p | AC091181.1 |
| 100 | hsa-miR-532-3p | AC015813.1 |
| 101 | hsa-miR-532-3p | AC134407.3 |
| 102 | hsa-miR-532-3p | AC114271.1 |
| 103 | hsa-miR-532-3p | AC092279.1 |
| 104 | hsa-miR-532-3p | LINC00662 |
| 105 | hsa-miR-532-3p | CEBPA-AS1 |
| 106 | hsa-miR-532-3p | AC005614.2 |
| 107 | hsa-miR-532-3p | LIPE-AS1 |
| 108 | hsa-miR-532-3p | NORAD |
| 109 | hsa-miR-532-3p | LINC00494 |
| 110 | hsa-miR-532-3p | AL132655.2 |
| 111 | hsa-miR-532-3p | AL118506.1 |
| 112 | hsa-miR-532-3p | URB1-AS1 |
| 113 | hsa-miR-532-3p | HLCS-IT1 |
| 114 | hsa-miR-532-3p | LINC01694 |
| 115 | hsa-miR-532-3p | AP001476.1 |
| 116 | hsa-miR-532-3p | AC002470.2 |
| 117 | hsa-miR-532-3p | NDUFA6-AS1 |
| 118 | hsa-miR-532-3p | AL031595.3 |
| 119 | hsa-miR-532-3p | LINC00685 |
| 120 | hsa-miR-532-3p | TSIX |
| 121 | hsa-miR-532-3p | LINC00894 |

Table S3. The immune-related signatures used in this study.

| **Immune-related signatures** | **Genes** |
| --- | --- |
| IMmotion150_Angio | VEGFA, KDR, ESM1, PECAM1, ANGPTL4, CD34 |
| IMmotion150_Teff | CD8A, EOMES, PRF1, IFNG, CD274 |
| IMmotion150_Myeloid | IL6, CXCL1, CXCL2, CXCL3, CXCL8, PTGS2 |
| JAVELIN | CD3G, CD3E, CD8B, THEMIS, TRAT1, GRAP2, CD247, CD2,CD96, PRF1, CD6, IL7R, ITK, GPR18, EOMES, SIT1, NLRC3, CD244, KLRD1, SH2D1A, CCL5, XCL2, CST7, GFI1, KCNA3, PSTPIP1 |

###
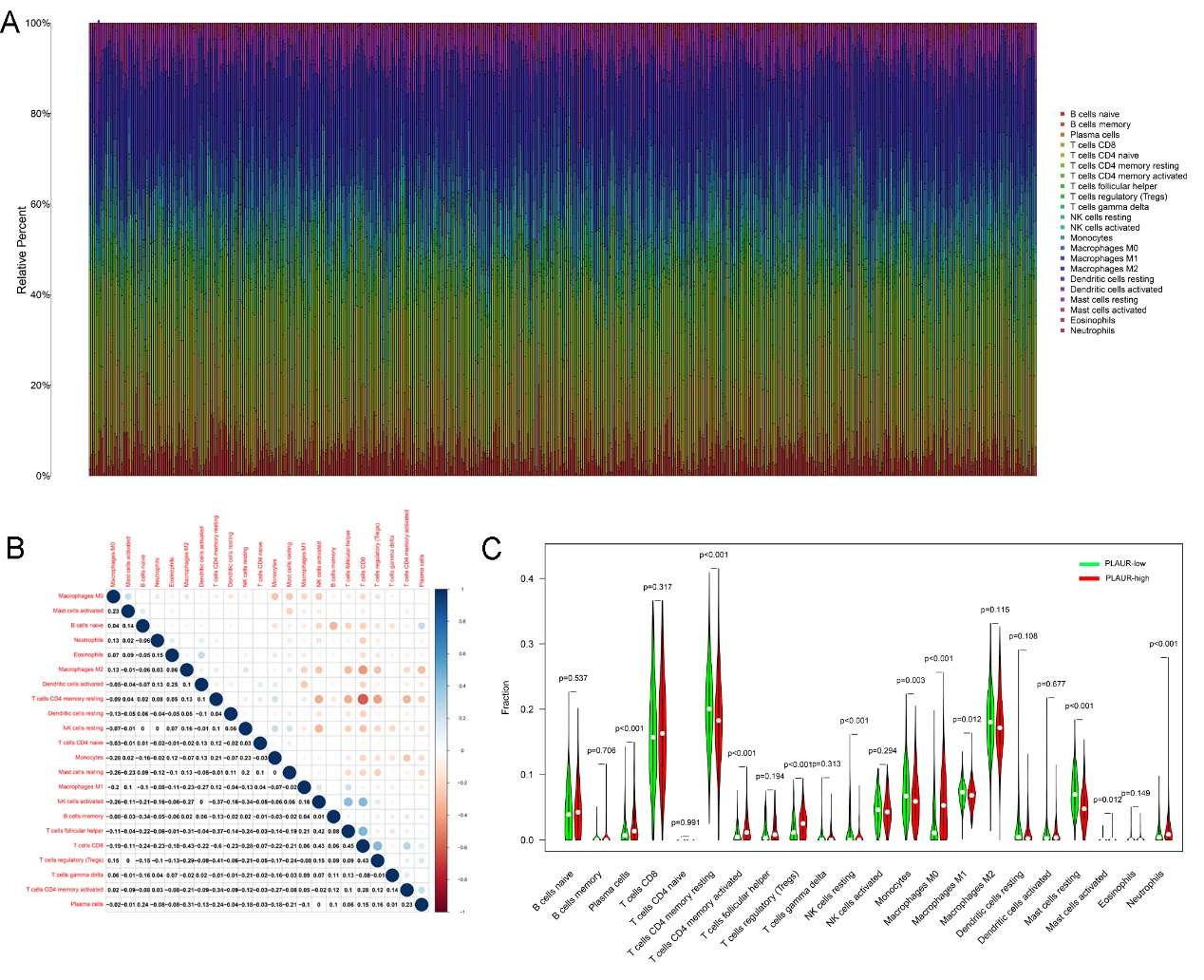


**Figure S1. Evaluation of the proportions of 22 types of immune cell infiltration by CIBERSORT algorithm based on the TCGA database.**(A) The landscape of infiltrating immune cells in KIRC. (B) Different correlation patterns among 26 immune cell subsets in KIRC. (C) Violin plots and heatmaps indicated the differences in the immune cell distribution between PLAUR low expression group and PLAUR high expression group in KIRC.


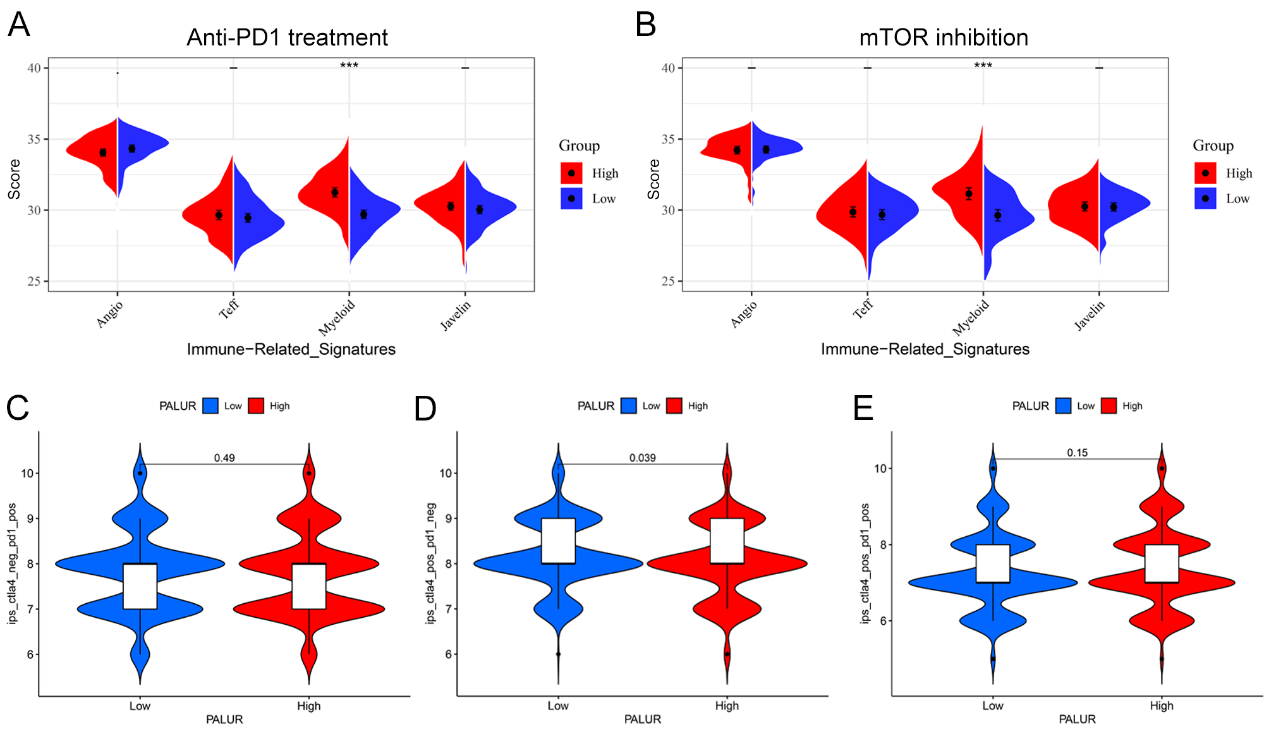


**Figure S2. Evaluation of the therapeutic responses by PLAUR.** (A) The difference of established immune signatures with response to anti-PD-1 therapy between low-PLAUR and high-PLAUR groups. (B) The difference of established immune signatures with response to mTOR treatment between low-PLAUR and high-PLAUR groups. IPS comparison between low-PLAUR and high-PLAUR groups stratified by CTLA4^neg^PD-1^pos^ (C), CTLA4^pos^PD-1^neg^ (D), and CTLA4^pos^PD-1^pos^ (E).


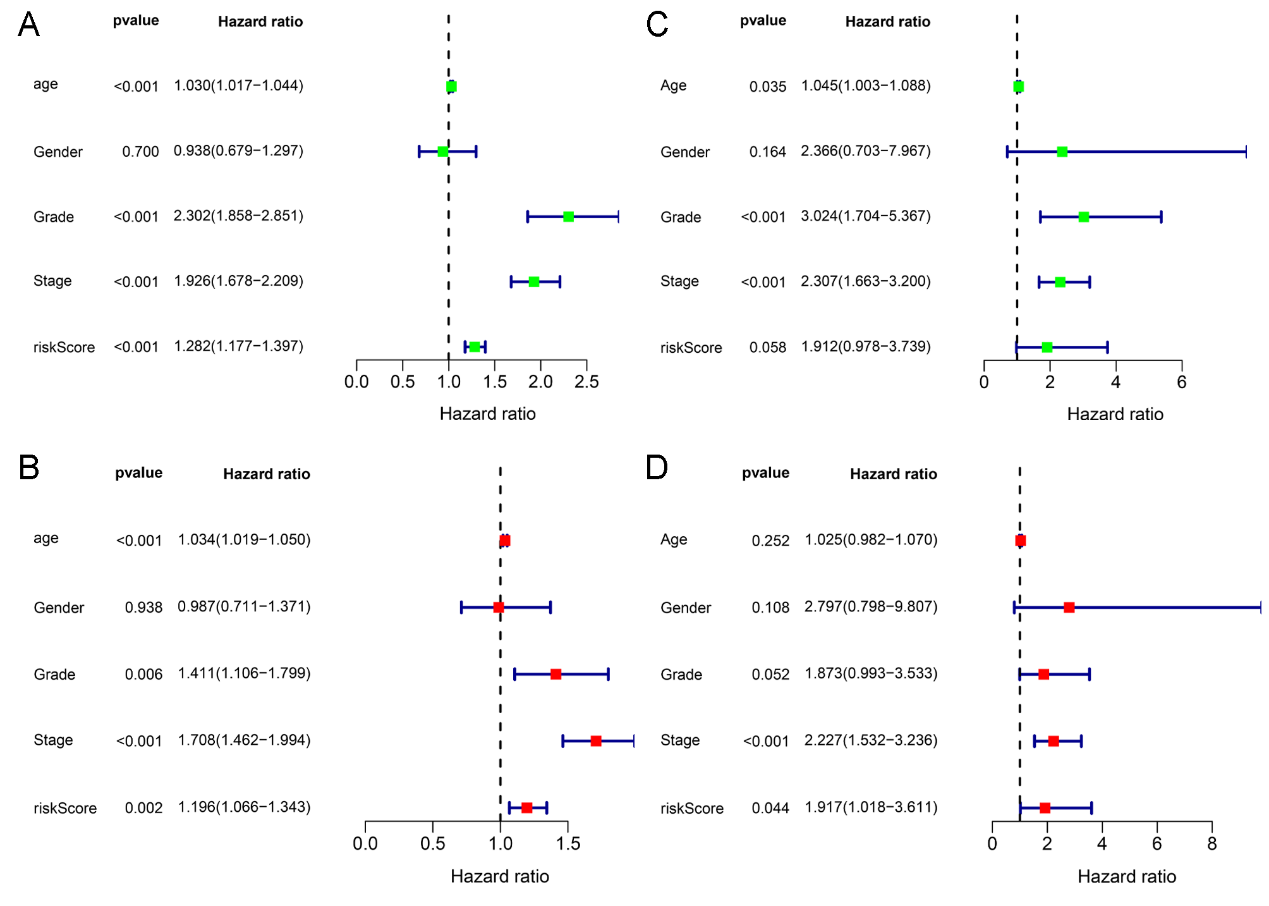


**Figure S3. Univariate and multivariate Cox regression analysis of clinical features with data from the TCGA cohort (A and B) and E-MTAB-1980 cohort (C and D).**
